# Antioxidant and Anti-Inflammatory Activities of the Extract and Bioaccessible Fraction of Mango Peel in Muffins

**DOI:** 10.17113/ftb.62.02.24.8258

**Published:** 2024-06

**Authors:** Yossaporn Plaitho, Aikkarach Kettawan, Hataichanok Sriprapai, Aurawan Kringkasemsee Kettawan, Phakpoom Kooprasertying

**Affiliations:** 1 School of Culinary Arts, Suan Dusit University, Sirinthon Road, Bang Phlat, Bangkok, 10700, Thailand; 2 Institute of Nutrition, Mahidol University, Phutthamonthon 4 Rd., Salaya, Phutthamonthon, Nakhon Pathom, 73170, Thailand; § These authors contributed equally to this work

**Keywords:** mango peel by-products, antioxidant activity, anti-inflammatory activity, health-promoting properties

## Abstract

**Research background:**

Mango peel is a production waste and can cause environmental problems, but its nutritional value consists of bioactive compounds that could be beneficial for human health. The aim of this study is to determine the bioactive compounds, antioxidant and anti-inflammatory activities of mango peels and their use in muffins.

**Experimental approach:**

The content of polyphenols, carotenoids and total phenols as well as the antioxidant activity of mango peel extract were evaluated. The anti-inflammatory activity of the extract was investigated using Caco-2 cell assay. The mango peel powder was then incorporated into muffin formulations. The sensory properties of these fortified muffins were evaluated. The total phenolic content, antioxidant activity and anti-inflammatory properties of the muffin extracts and their bioaccessible fractions were also analysed.

**Results and conclusions:**

The mango peel contained quercetin, phenolic compounds, α-carotene, β-carotene and lutein, which have antioxidant potential. In Caco-2 cells exposed to induced inflammation, the mango peel powder extract (*γ*=10, 50 and 100 µg/mL) attenuated the production of reactive oxygen species (ROS), tumour necrosis factor-alpha (TNF-α) and interleukin-8 (IL-8), while maintaining cell viability. Muffins supplemented with 5 % mango peel powder showed good sensory properties, but not as good as those of plain muffins without mango peel powder. The total phenolic content and antioxidant activities in both the extract and the bioaccessible fraction of the supplemented muffins were higher than those observed in the standard muffins. Moreover, the extract and bioaccessible fraction of the supplemented muffins resulted in a higher reduction of ROS, IL-8 and TNF-α production in Caco-2 cells than those obtained from the standard muffins.

**Novelty and scientific contribution:**

This study is the first to investigate the protective effects of mango peel and muffins supplemented with mango peel powder against IL-1β-induced oxidative damage in Caco-2 cells. The results confirm that both mango peel and the supplemented muffins inhibited the production of inflammatory markers, including ROS and cytokines. These findings suggest that mango peel could be a valuable component of functional food formulations including dietary supplements.

## INTRODUCTION

Thailand is a major mango-producing country and mangoes have an important role in the country’s economy. Mangoes can be consumed fresh or processed, *e.g.* into juices and purees or canned and dried. The consumption and processing of mangoes generates waste materials, including peels and stones, which are not usually consumed (*1*). Mango peels from processing are disposed of as waste and can cause environmental problems. Numerous studies have shown that mango peels have nutritional value that can be beneficial to humans as they contain antioxidants such as carotenoids, polyphenols and flavonoids, carbohydrates, proteins, fats, dietary fibre, calcium, zinc, iron and manganese (*2*, *3*), but they are not regularly consumed. However, most research focuses on the treatment of waste to extract bioactive compounds, a process that requires additional steps and generates other types of waste including chemical waste. Considering these facts, using mango peels as a functional food ingredient to develop functional foods not only serves to reduce waste, but also promotes the sustainable use of resources to support food security. Furthermore, it contributes to improved nutritional value and provides benefits for human health. Recently, mango by-products have been used in baked goods and food products such as pasta, noodles, tortilla chips, bread, biscuits and muffins (*4*). Besides mango peels, other by-products can be added to muffins, including goji berry by-products, grape by-products, pecan nut expeller meal, pomegranate peel and aniseed (*5*-*9*). However, research on the biological activities, specifically focusing on the antioxidant and anti-inflammatory properties, of food products integrating by-products such as mango peels and the biological activities of the bioaccessible fractions derived from these products, are still relatively limited.

Therefore, the aim of the present study is to investigate the effects of the proximate composition and bioactive compounds on the antioxidant and anti-inflammatory properties of mango peel powder and muffins containing mango peel powder. The results of this study could provide new insights into the use of mango peel for health purposes and also to reduce mango peel waste.

## MATERIALS AND METHODS

### Mango peel preparation

Fah-Lan mangoes (*Mangifera indica*) were collected from a local fruit market in Bangkok, Pathumthani and Suphanburi provinces, Thailand. The mangoes were washed twice with tap water and then peeled. The mango peels were dried with a towel and then cut into small pieces. These pieces were then further dried in a hot air oven (UF110; Memmert, Düsseldorf, Germany) at 50 °C for 8 h until their moisture content was reduced to less than 10 % (*3*, *10*). It should be pointed out that this drying negatively affects the antioxidant potential (*3*). The dried mango peel was ground and sifted through a 60-mesh sieve (V8SF; Gilson, Lewis Center, OH, USA).

### Proximate composition

Standardised methods from the Association of Official Analytical Chemists (AOAC) (*11*) were used to analyse the moisture, crude protein, crude fat, crude fibre, total sugar and ash mass fraction of the dried mango peel powder. The carbohydrate mass fraction (in %) was determined using the difference method, which calculates the remaining macronutrients after accounting for moisture, proteins, fats and ash:


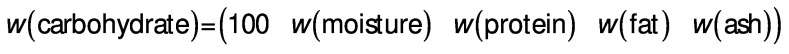
 /1/

### Determination of phenolic compounds

Briefly, a sample (10 g) was added to 40 mL of 62.5 % methanol solution, containing 0.5 g/L *tert*-butylhydroquinone (Sigma-Aldrich, Merck, St. Louis, MO, USA) and 10 mL of 6 M HCl (Merck, Darmstadt, Germany). After incubation in a water bath shaker (WTB50; Memmert, Germany) at 90 °C for 2 h the sample was cooled at room temperature. Then, 100 µL of 1 % ascorbic acid (Ajax Finechem, Victoria, Australia) was added and each sample was diluted to 50 mL with methanol (Merck). The solution was sonicated (5 min) followed by filtration through a 0.2 µm polytetrafluoroethylene (PTFE) syringe filter (Chrom Tech, Milford, MA, USA). The solution was analysed by high-performance liquid chromatography (HPLC) to quantify the following phenolic compounds: *p*-coumaric acid, caffeic acid, ferulic acid, sinapic acid, apigenin, hesperetin, kaemferol, luteolin, myricetin, naringenin, naringin, limonin and quercetin (Sigma-Aldrich, Merck). The HPLC system included a quaternary gradient pump (G1315A), a vacuum degasser (G1379A) and a Zorbax Eclipse XDB-C18 column (250×4.6 mm, 5 µm) with a matching guard column (Agilent Technologies, Santa Clara, CA, USA) maintained at 30 °C. The compounds were detected using a diode array detector (G1315B) at *λ*=210, 280, 325, 338 and 368 nm. The flow rate was adjusted to 0.6 mL/min with water (A), methanol (B) and acetonitrile (C), each containing 0.05 % trifluoroacetic acid. The solvent gradient was programmed as follows: 0–5 min (90–85 % A, 6–9 % B, 4–6 % C), 5–30 min (85–71 % A, 9–17.4 % B, 6–11.6 % C), 30–60 min (71–0 % A, 17.4–85 % B, 11.6–15 % C), 60–61 min (0–90 % A, 85–6 % B, 15–4 % C), and 61–66 min (90 % A, 6 % B, 4 % C) (*12*). Recovery rates for each standard ranged from 97 to 103 %, with mass fractions expressed in mg/100 g of sample.

### Determination of carotenoids

Briefly, the sample (10 g) was transferred to a brown round-bottom flask. Then, 10 mL of 10 % ascorbic acid (Ajax Finechem, Taren Point, NSW, Australia) and 50 mL of 2 M ethanolic potassium hydroxide (Ajax Finechem) were added. This solution was placed in a boiling water bath for 30 min and then cooled at room temperature. The sample was extracted with 70 mL of hexane (J.T. Baker, Phillipsburg, NJ, USA). After phase separation, the upper layer was decanted into an amber glass separating funnel containing 50 mL of a 5 % (*m*/*V*) potassium hydroxide solution. It was shaken and the lower layer was discarded. The sample was rinsed with 100 mL of water until it was free of alkali. Part of the solution was collected and evaporated under vacuum at 40 °C using a rotary evaporator (Buchi, Flawil, Switzerland). The sample residue was dissolved in methylene chloride (J.T. Baker, Phillipsburg, NJ, USA) and the mobile phase (1:2) before analysis. Carotenoid compounds, including α-carotene, β-carotene, β-cryptoxanthin, lutein, lycopene and zeaxanthin, were quantified using an Agilent 1260 Infinity system equipped with an ultraviolet-visible (UV-Vis) detector from Agilent Technologies. The carotenoids were separated using a C18 column (4.6 mm×250 mm, 5 µm, Vydac 201TP; Grace Davision, Waltham, MA, USA) equipped with a guard column (4.6 mm×12.5 mm, 5 µm, Vydac 201TP; Grace Division). The carotenoids were separated at a flow rate of 0.7 mL/min at 30 °C, and measured at *λ*=450 nm. The mobile phase was a mixture of HPLC-grade acetonitrile, methanol, methylene chloride, triethylamine and ammonium acetate (90:8:2:0.085:0.085, by volume) (*12*). The recovery rate for each standard compound was 96–102 %. The carotenoid mass fraction is expressed in mg/100 g of sample.

### Evaluation of the total phenolic content and antioxidant activity

#### Sample extraction

Sample was extracted using 90 % ethanol at a 1:10 (*m*/*V*) ratio and sonicated for 10 min in a sonicator bath (Mettler Electronics Corp, Anaheim, CA, USA). After sonication, the mixture was centrifuged (Dynac Centrifuge; Becton Dickinson, Sparks, MD, USA) at 5600×*g* and 25 °C for 10 min.

The supernatant was filtered and collected, while the residue was extracted twice with the same extract solution. The filtrates were combined and concentrated using a rotary evaporator under vacuum at 40 °C. The concentrated extract was dissolved again in deionised water for analysis (*13*).

#### Determination of the total phenolic content

In brief, 0.5 mL of extract, 8.0 mL of Folin-Ciocalteu reagent, 0.5 mL of distilled water and 1 mL of 20 % Na_2_CO_3_ were mixed and incubated in the dark (*14*). The absorbance was measured at 750 nm using a UV/Vis spectrophotometer (model UV-1601; Shimadzu, Chiyoda-ku, Tokyo, Japan). The total phenolic content was determined using a gallic acid calibration curve and is expressed in mg gallic acid equivalents (GAE) per g.

#### Evaluation of the 2,2-diphenyl-1-picrylhydrazyl radical scavenging activity

A volume of 22 μL of either sample extract or Trolox was mixed with 200 µL of 150 µM DPPH and incubated in the dark room for 30 min (*15*). The absorbance at 517 nm was measured using a microplate reader (Sunrise, Tecan Co., Grödi, Austria). Antioxidant activity was determined using a Trolox calibration curve and was expressed in µmol Trolox equivalents (TE) per g.

#### Evaluation of the Fe(III) reducing antioxidant power

A volume of 20 μL of either sample extract or iron(II) sulphate heptahydrate was mixed with 150 µL of FRAP reagent and incubated in the dark room for 8 min (*16*). The absorbance at 600 nm was then measured using a microplate reader (Sunrise, Tecan Co). The antioxidant activity was expressed in µmol Trolox equivalents (TE) per g.

#### Determination of the oxygen radical absorbance capacity

A volume of 500 μL of sample extract or 6.25–100 µmol Trolox was mixed with 3 mL of 4.19 µM fluorescein. The solution was incubated at ambient temperature for 8 min, then 0.5 mL of 153 mM 2,2-azobis(2-amidino-propane) dihydrochloride (AAPH) was added (*17*). Absorbance was measured at an excitation *λ*=495 nm and an emission *λ*=515 nm using luminescence spectrofluorometer (model LS 55; Perkin Elmer, Waltham, MA, USA). The antioxidant activity was expressed in µmol TE per g. A 75 mM phosphate buffer was used as a blank (control).

### Determination of anti-inflammatory activity using Caco-2 cells

#### Cell culture

Caco-2 cells were obtained from the American Type Culture Collection (ATCC, Rockville, MD, USA) and cultured in 6-well plates using Dulbecco's modified Eagle's medium (DMEM; Sigma-Aldrich, Merck) supplemented with 15 % heat-inactivated foetal bovine serum (FBS; Sigma-Aldrich, Merck), 1 % nonessential amino acids, 1 % l-glutamine, 0.2 % fungizone and 1 % penicillin-streptomycin (Gibco, Paisley, Scotland, UK) in a controlled environment (*φ*(air, CO_2_)=95 %) at 37 °C. The medium was replenished every 2 days, and the FBS amount was reduced to 7.5 % after 4–5 days of culture. Experiments were conducted using cultures aged between 11 and 14 days (*18*).

#### Cytotoxicity test

The cytotoxicity test was conducted using 3-(4,5-dimethylthiazol-2-yl)-2,5-diphenyltetrazolium bromide tetrazolium (MTT) assay. Caco-2 cells were washed with serum-free medium and then exposed to different concentrations (0–200 µg/mL) of mango peel extract at 37 °C for 24 h. After this incubation period, the cells were washed with phosphate-buffered saline (PBS), followed by the addition of 2.0 mL of MTT solution (0.5 mg/mL in PBS). The plate was further incubated at 37 °C for 4 h. After the incubation, the MTT solution was aspirated and the formazan crystals produced by viable cells were dissolved with dimethyl sulfoxide (DMSO). Absorbance was measured at 540 nm using a microplate reader (SPECTROstarNano, BMG LABTECH, Offenburg, Germany). If the absorbance values were above 90 % of the control samples, the sample was taken for further analysis (*19*).

#### Determination of anti-inflammatory activity

Caco-2 cells were cultured with mango peel powder extract in a controlled environment (*φ*(air, CO_2_)=95 %) at 37 °C for 1 h. Then, inflammation was induced by exposing the cells to 10 ng/mL interleukin-1β (IL-1β) at 37 °C for 30 min. The cells were harvested to evaluate reactive oxygen species (ROS) production using 2’,7’-dichloro-fluorescein diacetate (DCFH-DA) (*20*). In addition, the culture medium was collected to quantify tumour necrosis factor-alpha (TNF-α) and interleukin-8 (IL-8) levels using enzyme-linked immunosorbent assays (*21*).

### Evaluation of the characteristics of muffins supplemented with mango peel powder

#### Muffin production

The muffins were prepared with cake flour (200 g, UFM, Samut Prakan, Thailand), sodium hydrogencarbonate (4 g, McGarrett, Bangkok, Thailand), double-acting baking powder (8 g, McGarrett, Bangkok, Thailand), sour cream (40 g, Alli, Nakhon Ratchasima, Thailand), salt (2 g, Prungtip, Nakornratchasima, Thailand), pure vanilla extract (2.5 g, McCormick, Hunt Valley, MD, USA), egg (65 g, CP, Bangkok, Thailand), milk (125 g, CP-Meiji, Saraburi, Thailand), butter (50 g, Allowrie, KCG, Bangkok, Thailand) and granulated sugar (140 g, Mitrphol, Lei, Thailand). The egg was beaten before the milk, butter and sugar were added. After obtaining a smooth batter, the remaining dried ingredients were added and mixed. The muffins were baked at 180 °C for 20–25 min. Muffins were prepared with different additions of 5, 10 or 15 % mango peel powder, which replaced the same amount of the total flour.

The muffins were subjected to sensory evaluation using a 9-point hedonic scale and their colour, firmness and proximate composition were determined.

The sensory evaluation was done by 60 untrained panellists, consisting of 30 men and 30 women aged between 18 and 30, who had previously consumed muffins. Each sample was assigned a three-digit code. Each panellist received four samples with water to cleanse the palate between the samples. A 9-point hedonic scale ranging from 1 (representing "extremely dislike") to 9 (representing "extremely like") was used to evaluate the overall acceptability.

The colour of the muffins was evaluated in triplicate using a colorimeter (WF30; Fru, Shenzhen, PR China). Briefly, the colorimeter was placed on the surface of the muffin to measure the L* (lightness), a* (redness) and b* (yellowness) values.

The muffin texture, including hardness, springiness, cohesiveness and chewiness was measured with a texture analyser (TA.XT2i; Stable Micro Systems, Surrey, UK). Each sample was measured with a cylindrical shape probe (diameter 100 mm) in three different areas close to the centre. The muffin was cut into cylinders with *d*=2.65 cm and *h*=1 cm. The testing speed was set at 1 mm/s and the force applied was 25 % of the total height (*22*).

The nutritional value of the selected muffin was determined by proximate analysis (*11*). The energy amount was determined based on the components that provide energy (carbohydrates, proteins and fats).

### Quantification of total phenolic content, antioxidant and anti-inflammatory properties and bioaccessible fraction of the extract of muffins enriched with mango peel powder

Preparation of extracts of muffins containing mango peel powder

An extract of a muffin supplemented with mango peel powder was prepared as described above according to Muangnoi *et al.* (*13*). The total phenolic content, antioxidant capacity and anti-inflammatory properties were determined as described above.

#### *In vitro* digestion of muffins containing mango peel powder

The muffin sample (1 g) was mixed with 30 mL of 120 mM NaCl and then subjected to simulated gastric and small intestinal digestion according to Dawilai *et al.* (*23*) and Ferruzzi *et al.* (*24*). The pH of the sample solution was adjusted to 2.0 with 1 M HCl. The samples were then digested with 2 mL of pepsin in a shaking water bath at 37 °C for 1 h. The pH was adjusted to a range of 6.5 to 6.8 using 1 M sodium hydrogencarbonatebefore the addition of 3 mL of bile extract and 2 mL of pancreatin and 2 mL of lipase. The samples were shaken in a shaking water bath at 37 °C for 2 h. The digested (bioaccessible) fraction was separated by centrifugation (Dynac centrifuge; Becton Dickinson) at 10 000×*g* for 1 h and then filtered through 0.2-µm filter paper. The obtained sample was purged with nitrogen and stored at -80 °C until analysis.

### Statistical analysis

The data were collected in triplicate and statistically analysed to determine significance using *t*-test and ANOVA (*25*). Duncan's new multiple range test was used to evaluate differences among group means. Results are expressed as mean value±standard deviation (S.D.), with all assays conducted in triplicate for each independent treatment.

## RESULTS AND DISCUSSION

### Proximate composition, active substances and antioxidant potential of mango peel powder

Table 1 shows the proximate composition of the mango peel powder. It contained 84.8 % carbohydrates, of which 43.2 % was fibre. The findings corresponded closely to those reported in the investigations by Ajila *et al.* (*10*) and El-Faham *et al.* (*26*), who reported that carbohydrates, including crude fibre, were the dominant component of mango peel powder.

**Table 1:** The proximate composition, active substances and antioxidant activity of mango peel powder extracts

Component	*w*/%
Moisture	6.1±0.3
Carbohydrates	84.8±0.4
Crude fibre as part of carbohydrates	43.2±0.8
Total sugar as part of carbohydrates	13.0±0.3
Protein	4.0±0.1
Fat	1.17±0.09
Ash	3.9±0.2
Phenolic compound	*w*/(mg/100 g)
Quercetin	6.0±0.9
apigenin, caffeic acid, ferulic acid, hesperetin, kaemferol, limonin, luteolin, myricetin, naringenin, naringin, *p*-coumaric acid, sinapic acid	ND
Total phenols	*w*(GAE)/(mg/g)
	6.2±0.2
Carotenoid	*w*/(mg/100 g)
α-carotene	0.05?±0.001
β-carotene	2.3±0.9
Lutein	2.2±0.1
β-cryptoxanthin, lycopene, zeaxanthin	ND
Antioxidant activity	*b*(TE)/(µmol/g)
DPPH	3.72±0.01
FRAP	196.341.9
ORAC	85.7±1.1

The data are presented as the mean value±standard deviation (*N*=3). ND=not detected, GAE=gallic acid equivalents, TE=Trolox equivalents, ORAC=oxygen radical absorbance capacity

Of the phenolic compounds, only quercetin was detected, while other phenolic compounds could not be detected due to laboratory limitations. In contrast to a previous report (*27*), ferulic acid and caffeic acid were identified in raw Fah-Lan mango peel. The total phenolic content of mango peel extract in this study was lower than the data documented by Ajila *et al.* (*10*) and Pinsirodom *et al.* (*27*) (expressed as GAE on fresh mass basis, 9.59–9.86 mg/g).

Three carotenoids, namely α-carotene, β-carotene and lutein, were detected in the mango peel powder extract, while β-cryptoxanthin, lycopene and zeaxanthin were not detected. The result is in agreement with Ranganath *et al.* (*28*), who observed that β-carotene, lutein and α-carotene are the dominant carotenoids in mango peel powder extracts from a variety of cultivars (Hamle, Arka Anmol, Peach, BGNP Banganapalli, Janardhan Pasand, Lalmuni, Gulabi, Bombay No. 1, Lazzat Baksh and Tommy Atkins).

Table 1 shows the antioxidant potential of the powder from mango peel based on the DPPH, FRAP and ORAC assays. The antioxidant activity expressed as Trolox activity of mango peels from Sri Lanka showed a higher DPPH value (11.86–18.91 µmol/g) than in this study. In contrast, the FRAP value was lower (23.61–88.31 µmol/g) (*29*). Quercetin, lutein and β-carotene are widely recognised for their potent antioxidant properties attributed to their capacity to neutralise free radicals through electron or hydrogen donation mechanisms (*30*).

The differences in the proximate composition, active substances and antioxidant potential of mango peels might be due to the differences in the mango variety, the growing conditions (including fertilisation), climate, sample collection and preparation (*27*). Our data indicate that mango peels contain fibre, polyphenols and carotenoids, and they have antioxidant potential. Therefore, mango peels could be used as a supplement in healthy foods.

### The effect of mango peel powder extract on Caco-2 viability

Mango peel powder extract concentrations of 150 and 200 µg/mL were found to be toxic to Caco-2 cells, as evidenced by the fact that cell viability fell below 90 %. However, the other concentrations did not show any toxicity (Table 2). Therefore, concentrations of 10, 50 and 100 µg/mL were chosen for the following experiments.

**Table 2:** Cytotoxicity of the mango peel powder extract

*γ*(mango peel powder extract)/(μg/mL)	Cell viability/%
0	(100±0)
10	(98.7±3.1)^a^
50	(98.2±2.7)^a^
100	(96.8±3.4)^a^
150	(86.4±0.9)^b^
200	(85.7±2.2)^b^

The data are presented as the mean value±standard deviation (*N*=3). Different letters in superscript in the same column indicate a significant difference (analysis of variance followed by Duncan’s test, p<0.05)

### Anti-inflammatory activity of the mango peel powder extract

**Fig. 1:**
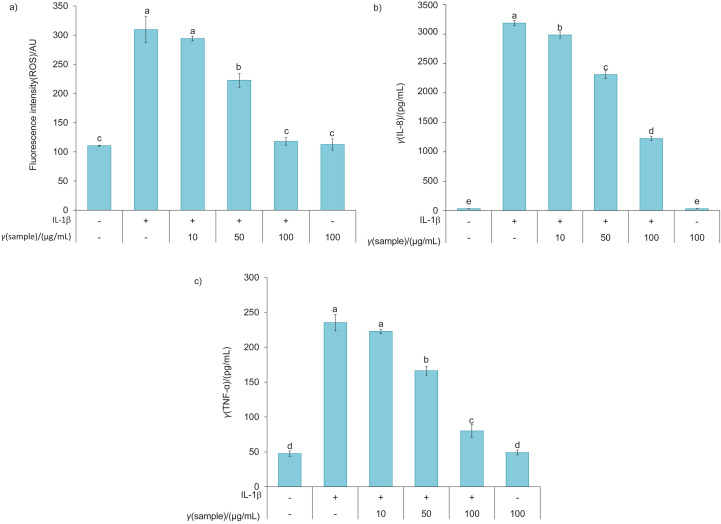
Suppression of IL-1β-induced production of: a) reactive oxygen species (ROS), b) interleukin-8 (IL-8) and c) tumour necrosis factor-α (TNF-α) by mango peel powder extract. Different letters indicate a significant difference (p<0.05)

Fig. 1 shows the anti-inflammatory effect of mango peel powder in Caco-2 cells. These cells were pretreated with mango peel powder extract (10, 50 and 100 µg/mL) for 2 h before stimulation with 10 ng/mL IL-1β for 30 min. This pretreatment resulted in a dose-dependent reduction in ROS, TNF-α and IL-8 production compared to Caco-2 cells treated with IL-1β alone, without an effect on cell viability (data not shown). Exposure of Caco-2 cells to mango peel powder extract (10, 50 and 100 µg/mL) significantly inhibited IL-8 secretion (6.35, 27.47 and 61.56 %, respectively). The concentrations of 50 and 100 µg/mL significantly inhibited the production of ROS (28.02 and 61.91 %, respectively) and TNF-α (29.31 and 65.93 %, respectively). These data indicate that the mango peel powder extract has an anti-inflammatory effect, perhaps because it contains quercetin, lutein and β-carotene, which have an anti-inflammatory activity. Previous studies have shown that pretreatment of PC-12 cells with quercetin significantly reduces intracellular ROS production when exposed to hydrogen peroxide (*31*). Kumar *et al.* (*32*) documented that diabetic retina treated with quercetin had a significantly lower TNF-α expression than untreated diabetic retina. Neelam *et al*. (*33*) showed that lutein administration reduced serum ROS levels in patients with non-proliferative diabetic retinopathy. In addition, lutein reduced in a dose-dependent manner the lipopolysaccharide-induced IL-8 secretion in uveal melanocytes (UM) (*34*). Moreover, Takahashi *et al*. (*35*) found that oral administration of β-carotene to mice reduced the inflammation and irritation associated with atopic dermatitis by suppressing the expression of TNF-α.

### Sensory and physical properties of muffins supplemented with mango peel powder

Table 3 shows the effect of the addition of mango peel powder to muffins on their sensory and physical properties. For the sensory evaluation, the standard muffins without mango peel powder obtained the highest scores for all characteristics. The addition of mango peel powder resulted in a significant reduction in sensory attributes (p<0.05). Among the supplemented muffins, those with 5 % mango peel powder had the highest sensory scores.

**Table 3:** Sensory evaluation using a 9-point hedonic scale and colour and texture analysis of standard muffins and muffins supplemented with different mass fractions of mango peel powder

	Score					
Parameter	Plain muffin		*w*(mango peel powder)/%			
			5	10		15
Sensory attribute						
Appearance	(7.8±0.4)^a^	(7.1±0.8)^b^		(6.6±0.6)^c^	(6.1±0.8)^d^	
Colour	(7.7±0.8)^a^	(7.2±0.9)^b^		(6.3±0.7)^c^	(6.2±0.7)^c^	
Odour	(7.5±0.8)^a^	(7.2±0.8)^b^		(6.6±0.6)^c^	(6.1±0.8)^d^	
Texture	(7.6±0.8)^a^	(7.1±0.8)^b^		(6.5±0.7)^c^	(6.2±0.7)^c^	
Taste	(7.6±0.8)^a^	(7.2±0.7)^b^		(6.7±0.5)^c^	(6.1±0.8)^d^	
Overall acceptance	(7.60±0.9)^a^	(7.2±0.6)^b^		(6.4±0.8)^c^	(6.2±0.4)^c^	
Colour						
L*	(60.8±0.7)^a^	(43.2±2.9)^b^		(34.4±0.7)^c^	(30.0±2.2)^d^	
a*^ns^	(6.0±2.1)	(8.0±0.6)		(8.7±1.0)	(9.5±4.4)	
b*	(12.1±3.5)^c^	(13.8±3.6)^bc^		(18.1±0.8)^b^	(23.0±0.4)^a^	
Texture						
Hardness/N	(388.2±6.9)^d^	(459.7±2.0)^c^		(545.9±3.3)^b^	(576.9±2.0)^a^	
Springiness/%	(63.2±3.6)^a^	(57.1±3.5)^ab^		(57.7±2.1)^ab^	(55.9±3.6)^b^	
Cohesiveness	(52.0±2.8)^a^	(50.0±1.0)^a^		(47.9±1.1)^ab^	(43.9±3.7)^b^	
Chewiness/N	(148.8+2.8)^b^	(171.5+6.1)^a^		(155.8+6.77)^b^	(136.6+5.8)^c^	

The data are presented as the mean value±standard deviation (*N*=3). Different letters in superscript in the same row indicate a significant difference (analysis of variance followed by Duncan’s test, p<0.05). ^ns^no significant difference

The amount of mango peel powder added to the muffins significantly affected the lightness and yellowness of the muffins. Similarly, the biscuits became darker with the higher amount of mango peel powder (*10*). Mango peel powder is brown due to enzymatic browning reaction occurring during mango peel powder processing, so adding it to muffins decreases the lightness of the product. The lightness of cakes also decreased with an increasing amount of potato peels (*36*). The b*, which indicates yellowness, increased gradually as the amount of mango peel powder increased. There was no significant difference for redness.

The mango peel powder also affected the textural properties of the muffins. Specifically, the hardness of the muffins increased as the amount of mango peel powder increased due to the high fibre content of this powder. *Vice versa*, as the amount of mango peel powder increased, the springiness, and cohesiveness of the muffins decreased. The findings are consistent with the results of Nakov *et al.* (*37*). The muffins with 5 % mango peel powder showed significantly different hardness and chewiness compared to the standard muffins. Thus, the most appropriate mango peel supplementation is 5 %, which represents approx. 1.5 % of the total muffin mass.

The muffins were also subjected to proximate analysis. The samples had similar fat and ash contents, but the crude fibre of muffins containing 5 % mango powder peel was higher than of the standard muffins (Table 4). Consistently, many researchers have found that by increasing the percentage of fruit by-products such as apple pomace (*38*), mango peel powder (*39*) and grape pomace (*37*), the total dietary fibre content in baked goods increases. The results indicate that mango peels can be an alternative source of fibre in baked goods.

**Table 4:** Nutritional value of plain muffins and muffins supplement with 5 % mango peel powder (100 g)

	Muffin		
Nutrient	Plain	Supplemented with mango peel	p
	*m*/g		
Carbohydrate	56.7±0.3	55.9±0.1	0.010
Crude fibre	0.25±0.03	0.81±0.02	0.000
Protein	5.75±0.03	5.49±0.01	0.000
Fat	11.0±0.2	10.7±0.1	0.165
Moisture	24.3±0.3	25.3±0.2	0.004
Ash	2.0±0.1	1.8±0.1	0.110
	*E*/kJ		
	1458.1±4.6	1431.1±6.4	0.004

The values are presented as the mean±standard deviation (*N*=3). The data were compared with an independent *t*-test (p<0.05)

### The total phenolic content and antioxidant potential of standard muffins and muffins containing mango peel powder

**Fig. 2:**
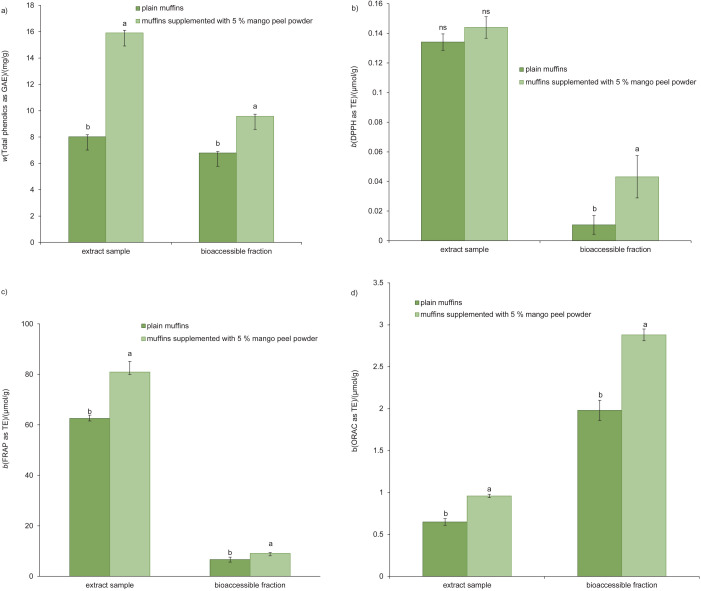
The total phenolic content (a) and DPPH (b), FRAP (c) and ORAC (d) activities of the extracts and bioaccessible fractions of plain muffins and muffins supplement with 5 % mango peel powder. Different letters indicate a significant difference (p<0.05). GAE=gallic acid equivalents, TE=Trolox equivalents, ORAC=oxygen radical absorbance capacity

Fig. 2 shows the total phenolic compounds and antioxidant potential of the extracts and bioaccessible fractions of standard muffins and muffins supplemented with 5 % mango peel powder. The total phenolic content, expressed as GAE, in the extract and bioaccessible fraction of the supplemented muffins ((15.9±0.2) and (9.6±0.2) mg/g, respectively) was significantly higher than the content in the standard muffins ((8.0±0.2) and (6.8±0.1) mg/g, respectively) (p<0.05).

The DPPH, FRAP and ORAC values, expressed as TE, were (0.14±0.01), (80.9±4.2) and (0.96±0.02) µmol/g, respectively, for the extract of muffins supplemented with 5 % mango peel powder, and (0.13±0.01), (62.5±1.3) and (0.65±0.04) µmol/g, respectively, for the extract of standard muffins. The extract of the supplemented muffins had significantly higher FRAP and ORAC values than the extract of standard muffins. There were no significant differences in the DPPH values (p>0.05) observed between the extracts of supplemented and standard muffins.

The bioaccessible fraction of muffins supplemented with 5 % mango peel powder had significantly higher antioxidant activity, expressed as TE ((0.04±0.01), (9.1±0.3) and (2.88±0.07) µmol/g for DPPH, FRAP and ORAC assay, respectively) compared to the bioaccessible fraction of standard muffins ((0.01±0.01), (6.6±1.0) and (2.0±0.1) µmol/g, respectively) (p<0.05). The total phenolic content of the bioaccessible fractions was lower than the total phenolic content of the extracts. Researchers have reported a lower total phenolic content after *in vitro* digestion of samples such as apples, pineapples, mangoes and papayas (*40*) as well as apples and apple snacks enriched with grape juice and coffee pulp (*41*) compared with undigested samples. This might due to interactions between phenolic compounds and digestive enzymes and other components such as buffers and electrolytes (*40*) or instability of quercetin during digestion (*42*).

The DPPH and FRAP values for the bioaccessible fractions were lower than the values for the extracts. Velderrain-Rodríguez *et al.* (*40*) and Khochapong *et al.* (*41*) reported comparable findings. However, the ORAC values of the bioaccessible fractions were higher than the values of the muffin extracts. Yida *et al*. (*43*) reported that at similar concentrations, digested edible bird’s nest had increased ORAC activity while undigested edible birds’ nest had little ORAC activity. Waisundara (*44*) also revealed that bioaccessible fractions of *Pouteria campechiana* pulp had higher ORAC activity than undigested sample, while the polyphenol content and DPPH activity were lower than the values in undigested samples.

### Anti-inflammatory activity of muffin extracts and bioaccessible fractions

**Fig. 3:**
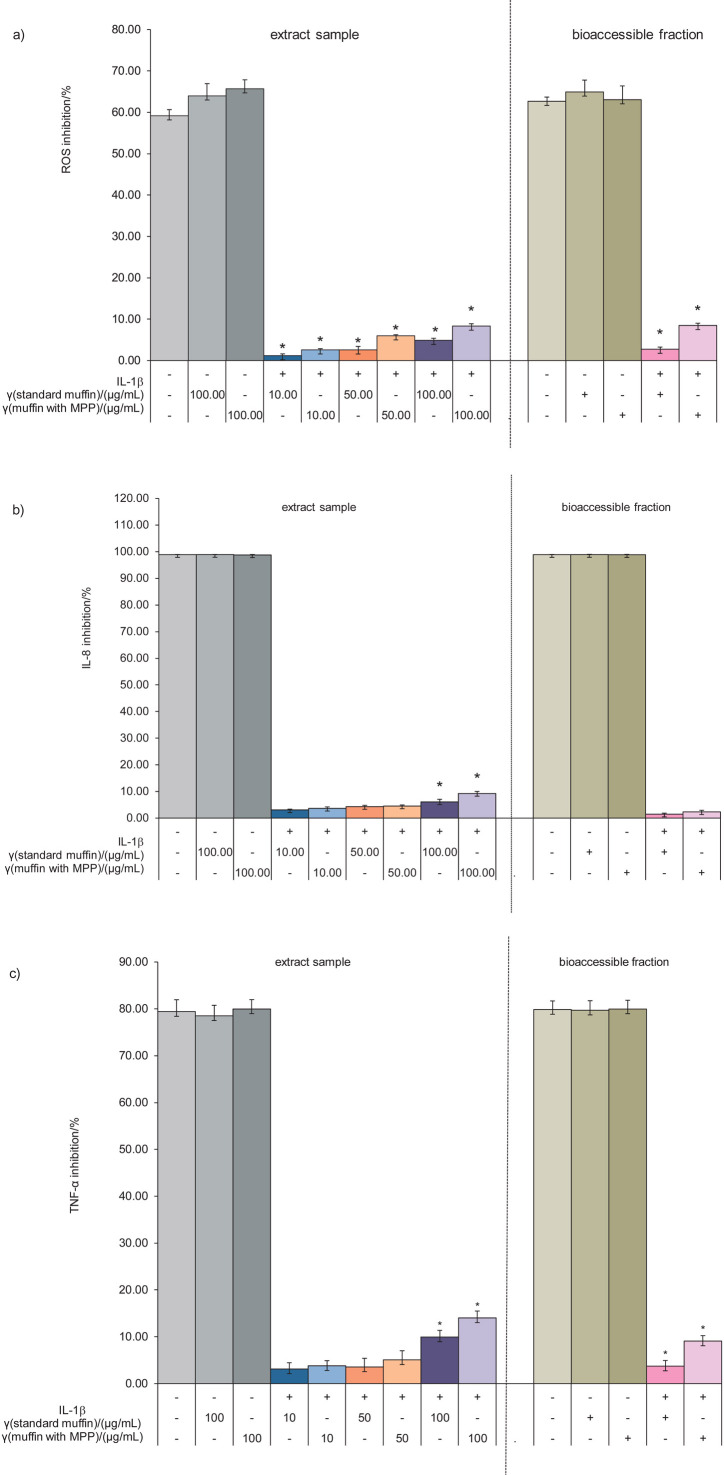
Percentage of inhibition of the extracts and bioaccessible fractions of plain muffins and muffins supplemented with 5 % mango peel powder of IL-1β-induced production of: a) reactive oxygen species (ROS), b) interleukin-8 (IL-8) and c) tumour necrosis factor-α (TNF-α). *Significant difference (p<0.05) between plain muffins and muffins supplemented with mango peel powder at the same concentration. MPP=mango peel extract

The anti-inflammatory activity results are expressed as the percentage of inhibition of inflammatory marker production (Fig. 3). Caco-2 cells were pretreated with muffin extracts (10, 50 and 100 μg/mL) or bioaccessible fractions for 2 h and then stimulated with 10 ng/mL IL-1β for 30 min. The standard muffin extract (10, 50 and 100 μg/mL) or supplemented muffin extract (10, 50 and 100 μg/mL) significantly reduced ROS production (1.19–4.88 and 2.58–8.33 %, respectively), IL-8 production (3.08–6.08 % and 3.65–9.23 %, respectively) and TNF-α production (3.12–9.95 % and 3.18–14.03 %, respectively). The bioaccessible fraction of standard muffins decreased ROS, IL-8 and TNF-α production by 2.74, 1.47 and 3.74 %, respectively. The bioaccessible fraction of supplemented muffins suppressed ROS, IL-8 and TNF-α production by 8.48, 2.34 and 9.09 %, respectively. The suppressed inflammatory marker production might be due to muffin ingredients such as egg, fresh milk and butter. Researchers have reported that whole egg consumption reduced TNF-α production in people with metabolic syndrome (*45*) and type 2 diabetes (*46*). Phosphatidylcholine is the predominate phospholipid found in eggs, which reduced the lipopolysaccharide-induced TNF-α secretion in a rodent model of systemic inflammation (*47*). Pasteurised cow’s milk reduced the expression of inflammatory cytokines such as IL-1, IL-6 and IL-8 in human gingival fibroblasts and human oral epithelial cell line HSC2 (*48*). Nestel *et al*. (*49*) showed that overweight adults who consumed butter had a lower secretion of inflammatory markers (Il-6, TNF-α and IL-1β).

Overall, the muffins supplemented with mango peel powder had better anti-inflammatory activity than the standard muffins, perhaps due to the quercetin, lutein and β-carotene content in mango peels.

## CONCLUSIONS

Mango peels contain many nutrients and beneficial substances, such as dietary fibre, carotenoids and polyphenols, which have antioxidant and anti-inflammatory properties. Moreover, mango peel extract has the potential to suppresses the production of inflammatory markers such as reactive oxygen species (ROS), tumour necrosis factor-α (TNF-α) and interleukin-8 (IL-8). Muffins containing 5 % mango peel received high consumer satisfaction scores, although they were still lower than for plain muffins. These muffins contained three times as much fibre as standard muffins. In addition, their extract and bioaccessible fraction had a greater antioxidant potential and aa stronger anti-inflammatory activity on gut cells than plain muffins. This is the first investigation of the protective effects of mango peels and muffins supplemented with mango peel powder against IL-1β-induced oxidative damage in Caco-2 cells.
